# Application and Multi-Stage Optimization of Daylight Polymer 3D Printing of Personalized Medicine Products

**DOI:** 10.3390/pharmaceutics14040843

**Published:** 2022-04-12

**Authors:** Jolanta Pyteraf, Adam Pacławski, Witold Jamróz, Aleksander Mendyk, Marian Paluch, Renata Jachowicz

**Affiliations:** 1 Department of Pharmaceutical Technology and Biopharmaceutics, Jagiellonian University Medical College, Medyczna 9, 30-688 Krakow, Poland; jolanta.pyteraf@uj.edu.pl (J.P.); adam.paclawski@uj.edu.pl (A.P.); aleksander.mendyk@uj.edu.pl (A.M.); renata.jachowicz@uj.edu.pl (R.J.); 2 A. Chełkowski Institute of Physics, University of Silesia in Katowice, ul. 75 Pułku Piechoty 1, 41-500 Chorzów, Poland; marian.paluch@us.edu.pl

**Keywords:** additive manufacturing, solid dosage forms, mebeverine hydrochloride, DoE, photopolymerization, personalized drugs

## Abstract

Additive technologies have undoubtedly become one of the most intensively developing manufacturing methods in recent years. Among the numerous applications, the interest in 3D printing also includes its application in pharmacy for production of small batches of personalized drugs. For this reason, we conducted multi-stage pre-formulation studies to optimize the process of manufacturing solid dosage forms by photopolymerization with visible light. Based on tests planned and executed according to the design of the experiment (DoE), we selected the optimal quantitative composition of photocurable resin made of PEG 400, PEGDA MW 575, water, and riboflavin, a non-toxic photoinitiator. In subsequent stages, we adjusted the printer set-up and process parameters. Moreover, we assessed the influence of the co-initiators ascorbic acid or triethanolamine on the resin’s polymerization process. Next, based on an optimized formulation, we printed and analyzed drug-loaded tablets containing mebeverine hydrochloride, characterized by a gradual release of active pharmaceutical ingredient (API), reaching 80% after 6 h. We proved the possibility of reusing the drug-loaded resin that was not hardened during printing and determined the linear correlation between the volume of the designed tablets and the amount of API, confirming the possibility of printing personalized modified-release tablets.

## 1. Introduction

Additive manufacturing technologies are an increasingly popular research subject related to the production of small batches of dosage forms according to patients’ needs [1,2,3]. These methods combine the advantages of large-scale industrial production and manufacturing of composed drugs in community or hospital pharmacies. They may become a flexible tool to produce small batches of drug delivery systems with personalized dosages of active pharmaceutical ingredients (API) [4,5,6]. Such a solution can be particularly important in the case of pediatric and geriatric populations, which often require dose adjustment based on the patient’s age and/or body weight. The potential of 3D printing technology to produce dosage forms with tailored drug release kinetics was confirmed by the Investigational New Drug (IND) approval from the U.S. Food and Drug Administration for the T19 product manufactured by Triastek to treat rheumatoid arthritis with the chronotherapeutic approach [7].

Three-D printing technologies can be categorized into three main groups: (i) methods based on extrusion, (ii) powder solidification, and (iii) liquid solidification, including photopolymerization [8]. Compared to other techniques, photopolymerization allows one to print objects without the use of heat such as selective laser sintering and fused deposition modeling, and can be applied for thermolabile drugs [4,9]. Due to the properties of the polymer matrix formed during the photopolymerization process, printed dosage forms can be characterized by extended-release of the API. However, the number of available photo-crosslinked polymers is limited, and these materials are not currently on the Generally Recognized As Safe (GRAS) list [9].

Techniques based on photopolymerization use photosensitive resins cured by light from a laser or light-emitting diodes (LEDs) [10]. As a building material, liquid resins containing monomers and photoinitiators are used, which induce a local polymerization reaction when stimulated with light of an appropriate wavelength. Since UV radiation is harmful to cells and tissues, the use of printers emitting visible light is preferable [11,12].

Up to now, some studies have been published on the manufacturing of dosage forms by photopolymerization-based techniques, including stereolithography (SLA) [13,14,15], digital light processing (DLP) [16,17,18], and liquid crystal display (LCD) [19]. The tested resin compositions contained monomers such as poly(ethylene glycol) dimethacrylate (PEGDMA) [20], N-vinyl-pyrrolidone [21], and poly(ethylene glycol) diacrylate (PEGDA) with different molecular weights [13,14,15,16,22,23]; and photoinitiators such as riboflavin [20,23], diphenyl(2,4,6-trimethylbenzoyl)phosphine oxide (DPPO) [13,14,15,16,21,22,23], and lithium-phenyl-2,4,6-trimethylbenzoyl-phosphinate [24]. Moreover, tested resins contained the addition of water; co-initiators such as triethanolamine [20,23]; or plasticizers such as poly(ethylene glycol) [13,16,21,22,23,24], glycerol, propylene glycol [21], or poly(caprolactone) triol [14].

In 2016, Wan et al. fabricated tablets with modified-release characteristics, in the first attempt to print dosage forms by stereolithography. The used resin was composed of PEGDA MW 700, PEG 300, DPPO, and an active ingredient: paracetamol or 4-aminosalicylic acid [9]. Karakurt et al. prepared controlled-release drug-loaded hydrogels. As a photocurable composition, they developed resin containing PEGDMA MW 550, 3% of triethanolamine, 0.1% of riboflavin as a photoinitiator, and ascorbic acid as a model active ingredient [20]. Moreover, Madzarevic and Ibric prepared and analyzed tablets made of a resin composed of PEGDA MW 700, PEG 400, water, riboflavin, and 5% ibuprofen as an API. They determined the influence of resin composition and printing settings (exposure time and wavelengths) on the drug dissolution rate from printed tablets [19].

In the case of Daylight Polymer Printing technology (DPP, also called liquid crystal display technology, LCD), light-emitting diodes (LEDs) are used as a light source, inducing the polymerization reaction. The LED system is located at the bottom of the printer and evenly illuminates the entire working area. The light emitted by the diodes is masked by an LCD screen placed directly below the resin tank containing a transparent fluorinated ethylene propylene (FEP) film on the bottom. As a result, the polymerization reaction during the printing of individual layers occurs only at points where the LCD screen does not mask the light from the LEDs [19,25].

Due to the non-availability of required dosages for personalized pharmacotherapy, the photopolymerization 3D printing method has real potential to be implemented for the preparation of small batches of extended-release tablets. However, this requires a further detailed analysis of numerous factors affecting the quality of the tablets, including resin composition, printing parameters, process repeatability, stability, and toxicity of resins, as well as printlets.

The aim of this study was the multi-stage optimization of a visible light-curable resin composition for printing drug-loaded tablets by the daylight polymer printing method. Formulations containing varying amounts of water, PEG 400, PEGDA MW 575, and riboflavin as a photoinitiator were tested based on the full factorial design of the experiment (DoE) to determine the best resin composition characterized by good mechanical properties after the curing process. Next, we analyzed the influence of the printer modifications and the addition of co-initiators ascorbic acid or triethanolamine on the tablets’ properties, resin reusability, and required printing time. The optimized resin composition was used to print drug-loaded tablets containing mebeverine hydrochloride as a model drug.

## 2. Materials and Methods

### 2.1. Materials

Poly(ethylene glycol) diacrylate with molecular weight 575 (PEGDA) and poly(ethylene glycol) with molecular weight 400 (PEG, both from Merck^®^ KGaA, Darmstadt, Germany) were used as resins, forming monomer and plasticizer, respectively. Riboflavin (vitamin B2, Sigma-Aldrich, Saint Louis, MO, USA) was used as a photoinitiator. Ascorbic acid (AscA, Fagron sp. z o.o., Cracow, Poland) and triethanolamine (TRI, Avantor Performance Materials Poland S.A., Gliwice, Poland) were added to the formulation as co-initiators, while mebeverine hydrochloride (MEB, Benechim SPRL, Lessines, Belgium) was used as a model active ingredient. Water used in all experiments was produced by an Elix 15UV Essential reversed osmosis system (Merck^®^ KGaA, Darmstadt, Germany).

### 2.2. Optimization of Formulation

The optimization of the formulation included the following steps:

1. Evaluation of PEGDA/PEG/water ratio: different amounts of PEGDA MW 575, PEG 400, and water with 0.1% addition of riboflavin were tested in PVC molds to optimize photosensitive resin curable with visible light. The compositions of the tested formulations were selected based on the full factorial design of the experiment.
2. 3D printing of placebo tablets and printer customization: optimized resin was used to print placebo tablets using a Daylight Polymer Printing technology. To improve the quality of the printed tablets and reduce the amount of resin needed for printing, an overlay on the printer’s platform and a smaller resin tank with an adapted holder were used.
3. Assessment of the impact of co-initiators: the best resin composition was used to evaluate the effectiveness of ascorbic acid and triethanolamine to improve the mechanical properties of the tablets and reduce the printing time.
4. Evaluation of the possibility of reusing the uncured resin left over from printing.
5. 3D printing of drug-loaded tablets: process optimization and analysis.

### 2.3. Evaluation of PEGDA/PEG/Water Ratio

In order to establish the optimal composition of a resin for the 3D printing process, the full factorial design of the experiment was planned and executed. Analyses of the DoE plan and further results were conducted using the R [26] environment with additional package rsm v.2.10.3 [27,28]. Two factors at three levels were included in the DoE (3^2^ design), resulting in 11 experiments, including 9 unique compositions (P1–P9) presented in Table 1. Moreover, we prepared two additional repetitions for the P5 resin composition (marked sequentially as P5′ and P5″) to acquire information about the variability of the outcome variable in the applied production process and the product’s assessment methodology. Within analyzed factors, PEGDA content was: 15% (*w*/*w*), 45% (*w*/*w*), and 75% (*w*/*w*), encoded as levels −1, 0, and 1, respectively. The second factor, water content, was varied between 5% (*w*/*w*), 15% (*w*/*w*), and 25% (*w*/*w*) and was encoded as levels −1, 0, and 1, respectively. The third compound, PEG 400, was added as needed to the total amount of 99.9% (*w*/*w*). All tested resins contained 0.1% (*w*/*w*) of riboflavin, used as photoinitiator. Experiments were executed in random order to eliminate the effect of the sequence of factors on the obtained results. The full DoE setup is represented in Table 1.

A total amount of 10 g of each resin was prepared by mixing its components in a glass beaker and dispensing 400 µL into five PVC blister slots (approx. 10 mm × 25 mm) used as a mold. Next, the dispensed resins were exposed for 30 min to visible light with a wavelength of 460 nm in a Liquid Crystal PRECISION 1.5 3D printer (Photocentric Ltd., Peterborough, UK). After exposure to the light, cured tablets were washed using ethanol to remove the residual part of uncured resin. Next, a penetration test was performed on all of the cured resin samples to measure the force required to break them.

### 2.4. Mechanical Properties

The mechanical properties of all cured resins and printed tablets were analyzed using a penetration test. The test was performed with an EZ-SX tensile tester (Shimadzu, Kyoto, Japan) equipped with an indentation test jig (needle probe). A pre-test procedure was performed: the probe was lowered at 5 mm/min until it reached the surface and put pressure of 0.001 N on the tested material, then the test began. During the test, the needle probe was dipped into the cured resin/tablet at a speed of 2 mm/min, and the force needed to break the structure was measured.

### 2.5. Model-Driven Optimization of Resin Composition

Based on the 3-level full factorial design (DoE), first and second-order predictive models were developed with the further objective of process optimization. The dependent variable was hardness of the tablets, and as independent variables, DoE factors were included. The response surface method (RSM) was used for final assessment of the optimal settings for resin composition in regard to the expected hardness of the tablets.

### 2.6. 3D Printing of Placebo Tablets

The resin containing optimized quantities of PEGDA, PEG, and water was used to prepare the first batches of tablets. The project of a round tablet with 10 mm diameter and 5 mm of height was created in the Blender 2.80 3D modeling software (Blender Foundation, Amsterdam, The Netherlands). Next, specific files for the 3D printer were prepared using Photocentric Studio 1.0.2.9 slicing software (Photocentric Ltd., Peterborough, UK) with customized printing profiles as presented in Table 2. The adjustment of the printing profiles included changes in the height of the layers and exposure time to print tablets characterized by optimal mechanical properties. To obtain a monolithic tablet structure, the exposure time was the same for all layers (without extending the exposure time of the bottom layers used for this method). The names of the following series of tablets were created based on the type of tablet (e.g., “TP” for placebo tablets), an additional component (“AscA” or “TRI”, if used), and information about printing parameters: the layer height and exposure time (e.g., 100/200).

Each time, 60 g of resin was prepared; the resin components were placed in an amber glass jar, mixed for 15 min with a magnetic stirrer, and poured into a resin tank of the printer. Tablets were fabricated using the Liquid Crystal Precision 1.5 3D printer (Photocentric Ltd., Peterborough, UK). The printer’s construction contained the LED system light source, masked by the LCD screen responsible for displaying the outlines of individual layers of the object. After printing, tablets were washed out from residual uncured resin, weighed, measured, and tested using a texture analyzer. The first series of tablets, encoded as TP_100/200, was printed with the following printer settings: 100 µm of layer height and 200 s of exposure time for a single layer. Next, different layer height and exposure time settings were tested to improve the tablets’ mechanical properties and mass uniformity (Table 2).

### 2.7. Assessment of the Impact of Co-Initiators

In order to shorten the printing time and improve the quality of the tablets (uniformity of mass and dimensions), in subsequent tests we assessed the effect of the addition of co-initiators: ascorbic acid or triethanolamine. A suitable amount of co-initiator, 1.0, 0.5, or 0.1% (*w*/*w*) for ascorbic acid or 0.15% (*w*/*w*) for triethanolamine, was included in the optimized placebo resin containing a correspondingly reduced amount of PEG. The method of preparing the resins was the same as in the previous stage. Composition and printing parameters, presented in Table 2, were modified to achieve the shortest printing time while maintaining acceptable properties of printed tablets.

### 2.8. Evaluation of Resin Reusability

To evaluate the reusability of resin, we designed an experiment to reuse the liquid resin that remained in the resin tank after printing of the tablets. The resins remaining after printing the TP_50/100 and TP_TRI_50/25 series of tablets were strained after printing using 400-micron paint strainers and stored at room temperature in an amber glass jar for 24 h. Directly before the second printing process, resins were mixed for 15 min with a magnetic stirrer. All printer settings were the same as during the first trial. As a result, we prepared and analyzed two series of tablets marked as TP_50/100′ and TP_TRI_50/25′, made of resins remaining after printing of the original TP_50/100 and TP_TRI_50/25 tablets, respectively (Table 2).

### 2.9. Preparation of Drug-Loaded Tablets

The MEB-loaded tablets were made of the optimized placebo resin formulation containing co-initiator. Each time, 60 g of resin was prepared by mixing 54 g of placebo resin and 6 g of mebeverine hydrochloride in an amber glass jar with a magnetic stirrer for 12 h. The MEB-loaded resins were used to print 4 series of 14 round tablets with a diameter of 10 mm and a height of 5 mm with the settings presented in Table 3. After printing, the resins were mixed and used to print the second series of tablets with the same printing parameters (series TMEB_50/25′ and TMEB_50/50′, respectively). Fresh resins were prepared for the tests with different printing settings. After the printing processes, tablets were washed from the residual part of uncured resin and left at room temperature protected from light for 24 h to dry.

Differences between the series of tablets made of the new and reused resin were evaluated with Student’s *t*-test using the R environment [26]. The test assumptions, including normality and homogeneity of variance, were confirmed using Shapiro–Wilk and F tests, respectively.

### 2.10. Drug Content in Tablets

Three randomly selected tablets from each series, TMEB_50/25, TMEB_50/50, and TMEB_50/50′, were analyzed. Accurately weighed samples were placed in conical flasks filled with 50 mL of water and shaken for 24 h in a Memmert^®^ water bath (WNB 22, Schwabach, Germany) at 37 °C. Next, samples were filtered through CHROMAFIL^®^ Xtra CA-45/25 syringe filters. The concentration of MEB was determined spectrophotometrically at 264 nm using a Jasco V-530 spectrophotometer (Tokyo, Japan). The specificity of the analytical method was verified; there was no significant interference between the drug and excipients at the analytical wavelength. The linearity was confirmed in the range from 10 to 60 µg/mL (R^2^ = 0.99993).

### 2.11. Dissolution Study

The release of mebeverine hydrochloride from 3D printed tablets was evaluated in 1000 mL of water using a USP-II apparatus (Vision^®^ G2 Elite 8, Hanson Research^®^, Chatsworth, CA, USA) equipped with a VisionG2 AutoPlus autosampler. The samples were withdrawn at predetermined time points, filtered, and assayed spectrophotometrically at 264 nm using a UV-1800 spectrophotometer (Shimadzu^®^, Kyoto, Japan) equipped with flow-through cuvette. Six repetitions for each sample were carried out. The results represent the averaged amount of API released and the standard deviation (mean ± SD).

For comparation purposes, compounded gelatin capsules containing mebeverine hydrochloride were prepared. The amount of MEB equivalent to the API content in the 3D printed tablets was distributed manually into 6 size-3 capsules (ACG Lukaps d.o.o., Ludberg, Croatia; 48 mg of MEB in each capsule). To avoid flotation during the dissolution study, the capsules were placed in pharmacopoeia basket sinkers (according to European Pharmacopoeia, Ph. Eur., 10th Edition) [29].

In order to establish the mechanism of drug dissolution from the 3D printed dosage form, KinetDS 3.0 software was used [30]. The software offers an automatic fitting procedure for the most popular mechanistic and empirical models applied to the drug dissolution curve description. Models are assessed by the coefficient of determination (Equation (1)) and root-mean-square error (RMSE, Equation (2)).

(1) $$R^{2}=1-\frac{SS_{res}}{SS_{tot}}=1-\frac{\sum_{i=1}^{n}{\left(obs_{i}-pred_{i}\right)}^{2}}{\sum_{i=1}^{n}{\left(obs_{i}-obs_{mean}\right)}^{2}}$$

where: R^2^ is coefficient of determination, *SS_res_* is the sum of squares of the residual errors, *SS_tot_* is the total sum of the errors, *obs_i_*, *pred_i_* are observed and predicted values, and *obs_mean_* is the arithmetical mean of observed values.

(2) $$\mathrm{RMSE}=\sqrt{\frac{\sum_{i=1}^{n}{\left(obs_{i}-pred_{i}\right)}^{2}}{n}}$$

where: *obs_i_*, *pred_i_* are observed and predicted values, *i* is the data record number, and *n* is the total number of records.

### 2.12. Project Volume–Dosage Correlation

To assess the correlation between the volume of designed tablets and the dose of API, a series of tablets with different volumes were printed and analyzed. For this purpose cylindrical tablets with heights of 3, 4, 5, 6 or 7 mm and twice the diameter were designed. Projects were used to print tablets made of an optimized drug-loaded resin, with a printing profile the same as for TMEB_50/50 tablets, in triplicate for each volume.

## 3. Results and Discussion

### 3.1. Optimization of Formulation

**Figure 1:**
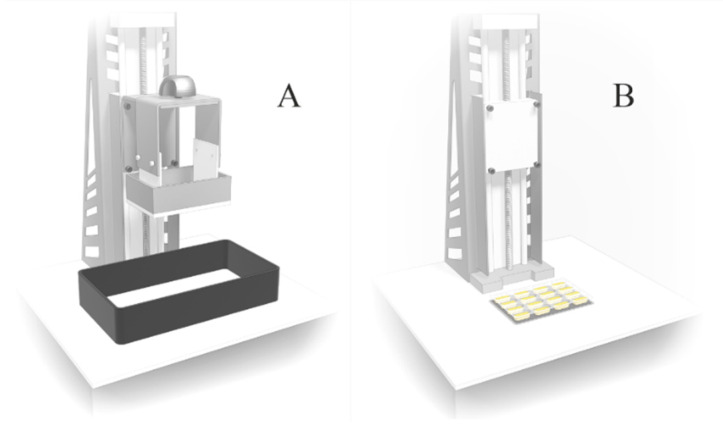
3D printer structure: (**A**)—original; (**B**)—adapted to cure resin in blisters.

**Figure 2:**
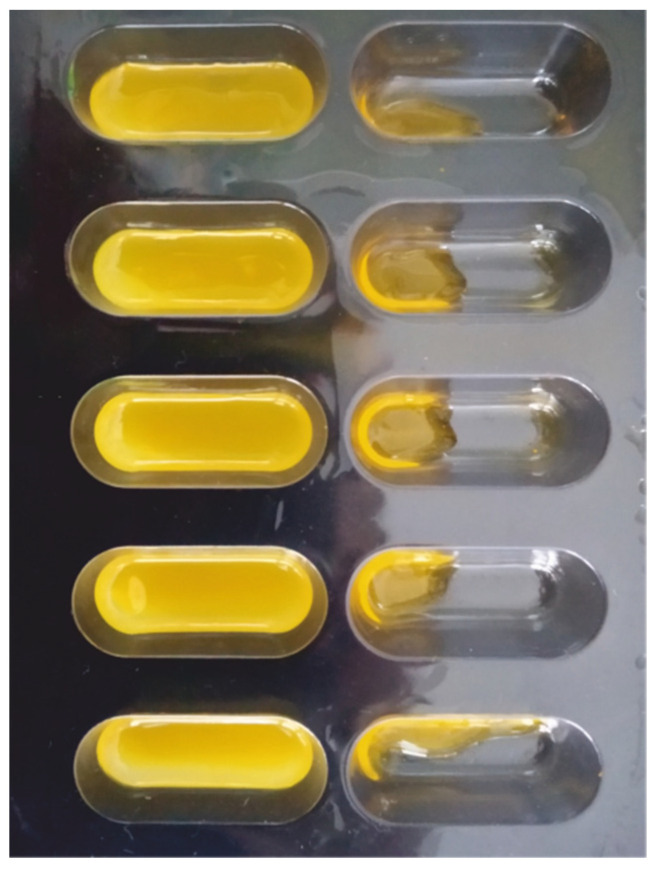
Examples of cured (**left** side) and uncured (**right** side) resins in blisters after 30 min of exposure to visible light and rinsing out the residual resin.

In order to reduce the amount of resin necessary to perform the pre-formulation stage, we developed a method of curing small resin samples in the blister slots, which replaced the resin tank (Figure 1). The use of blisters as forms for irradiating resins decreased the amount of resin necessary for the tests from 100 g for standard printing to only 10 g for each composition. In addition, it was easier to observe the boundary printability properties. In the case of printing using resins with non-optimal properties, the tablets would be torn off the printer’s platform and hardened to a shapeless mass, difficult to analyze. Placing the resins in the blister slots and extending the exposure time allowed to produce a solid or semi-solid mass made the measurement and comparison of mechanical properties possible. Only in the case of formulations P3 and P9, the resin was uncured after exposure to the light, and the analysis was impossible (Figure 2). The results of mechanical tests of cured resins used in formulation optimization, based on the values of the forces needed to break the hardened resins, are presented in Table 4. For uncured resins (P3 and P9), force values were assumed as 0.

**Figure 3:**
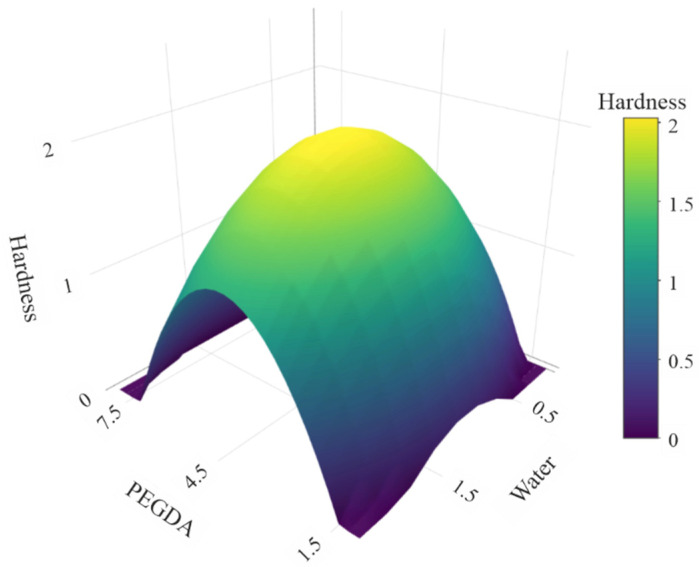
Response surface of developed second-order model.

The results obtained from experiments were applied to develop predictive models employed to find the optimal composition of resin. Two types of empirical models—first-order and second-order models—were developed using the R environment [26]. The first type assumed only linear relationships between factors and response, whereas the second one allowed for identification of nonlinear relationships. The models were assessed on the basis of the statistical significance of predictions (*p*-value) as well as goodness-of-fit parameters such as residual standard error (RSE) and adjusted R-squared. The summary of the models’ analysis is presented in Table 5. The first-order model was not statistically significant (*p*-value = 0.93) and presented poor fitting to the data expressed in RSE = 1.01 and adjusted R^2^ = −0.23. The second-order model was found to be statistically significant (*p* < 0.005) and presented a better fit to the data: RSE = 0.32 and adjusted R^2^ = 0.88. Besides intercept, just coefficients for X1^2^ and X2^2^ were significant, pointing to a nonlinear relationship between examined factors and the tablet’s hardness. The second-order model was later applied to find the optimal resin composition in terms of the highest hardness of produced tablets. The analysis was performed by generating a grid of points probing PEGDA and water content within the original design range and in 0.25 g increments. Predictions of the model represented as a response surface are presented in Figure 3. Based on the numerical experiment, the optimal composition was established as PEGDA = 45% (*w*/*w*), water content = 15% (*w*/*w*), 0.1% (*w*/*w*) of riboflavin, and 39.90% of PEG. Surprisingly, the optimal resin composition predicted by the model was found to be the P5 original DoE composition.

### 3.2. 3D Printing of Placebo Tablets and Customization of the 3D Printer

**Figure 4:**
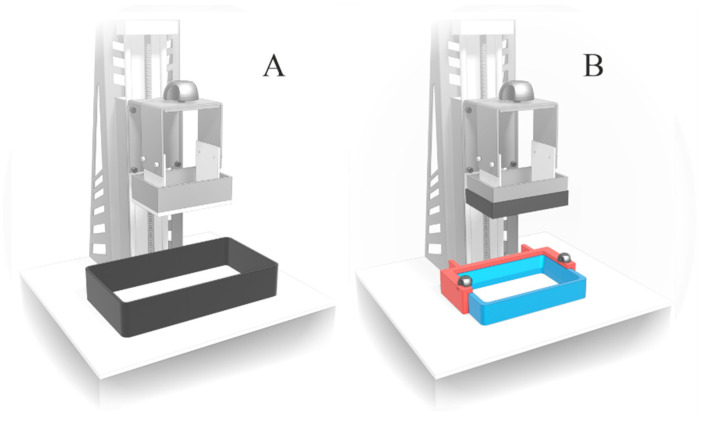
3D printer structure: (**A**)—original; (**B**)—adapted: an overlay on printer platform (black), replaced resin tank with handle (marked blue and red, respectively).

**Figure 5:**
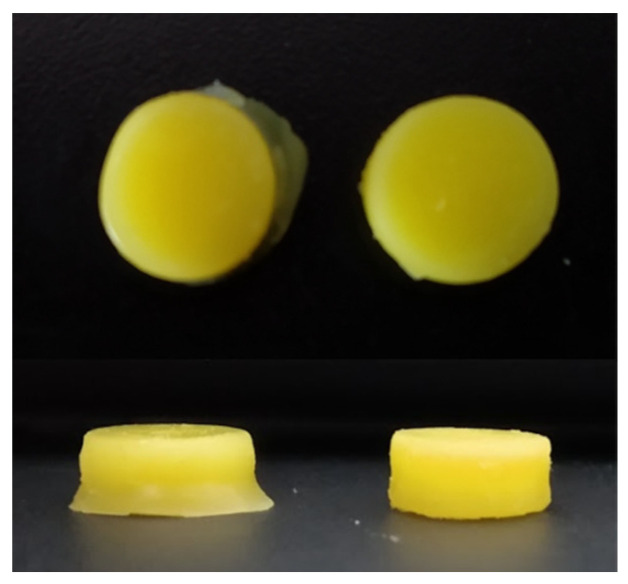
Tablets printed on original (**left** side) and modified printer platform (**right** side).

The first series of tablets was made of the optimized placebo resin composition, with the following printing settings: 100 µm of layer height and 200 s of layer exposure time. During the process, 18 tablets distributed at equal intervals on the printer’s platform were printed simultaneously. Printing succeeded, but we observed an increase in the diameter of the tablets at the first layers, which could have been an effect of the reflection of light by the aluminum printer’s platform. In the case of subsequent layers distant from the platform, this effect was not noticeable. Therefore, we prepared an overlay on the platform, made of black PET (Polyethylene terephthalate), using a ZMorph VX Fused Deposition Modeling 3D printer (Wroclaw, Poland). Additionally, we replaced the original printer resin tank with a smaller one (Anycubic, Shenzhen, China), with a PET-made custom-printed handle stabilizing its position during printing. The above-mentioned modifications, presented on Figure 4, reduced the amount of resin needed for printing a series of tablets from 100 g to only 50 g. Additionally, we observed a minimization of tablet side edge deformation (Figure 5).

Despite the modification of the printer, the diameter of TP_100/200 tablets was 8% larger in comparison to the project (Table 6). Additionally, the tablets’ mass was not uniform. Thus, we reduced the height of the layer while maintaining the total exposure time to increase the accuracy of the dimensions of the tablets. Despite the long exposure time for the TP_50/100 series, the tablets’ diameter was smaller than theoretical. The tablets’ mass variations were even higher in comparison to those with the previous settings. Moreover, the reuse of resin used once for printing was impossible. During the attempt to use the uncured resin remaining after printing, only small and irregular points were hardened instead of tablets (Table 6, TP_50/100 and TP_50/100′ for printing using fresh and reused resin, respectively). Due to the low reproducibility and necessity of preparing fresh resin for each series of tablets, we assessed the effectiveness of additional compounds to improve the resin’s properties.

### 3.3. Co-Initiator Impact Assessment and Optimization of Printing Settings

Riboflavin (vitamin B2) is a naturally occurring, water-soluble orange-yellow powder with absorption maxima in the visible and UV ranges [31]. It is a “generally recognized as safe” (GRAS) substance, and is excreted with urine within a few hours [32]. Riboflavin is a type II photoinitiator that requires an electron donor such as tertiary amines or ascorbic acid to generate a free radical upon light irradiation [33,34]. Thus, two types of co-initiators were tested: ascorbic acid and triethanolamine.

**Figure 6:**
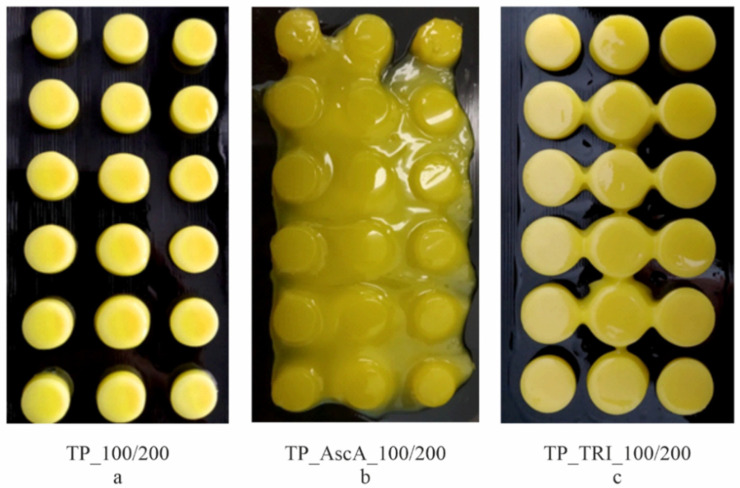
Placebo tablets made of different compositions: without a co-initiator (**a**), with ascorbic acid (**b**), with triethanolamine (**c**), printed with 100/200 printing profile.

A photosensitive formulation composed of a bioactive form of riboflavin and ascorbic acid for reversible addition–fragmentation chain transfer (RAFT) polymerization had been developed by Zhang et al. They analyzed a two-component initiation system, which was able to initiate under blue LED irradiation the controlled RAFT polymerization of acrylamides, acrylates, and methacrylates [34]. In the first stage, we tested resin containing 1% of ascorbic acid with the following printing parameters: 100 µm of layer height and 200 s of exposure time. The addition of ascorbic acid caused the resin to harden over a larger surface and produce a fixed, shapeless form with clearly marked tablets (Figure 6b). Therefore, in subsequent trials, we reduced the amount of ascorbic acid and used concentrations of 0.5% and 0.1%, respectively. Regardless of the amount of ascorbic acid used, the effect was similar. Due to the irregular cured surface and the inability to print tablets with sharp edges, printing attempts from resins containing ascorbic acid were considered unsuccessful.

Next, we tested formulations containing 0.15% (*w*/*w*) of triethanolamine. The concentration of TRI was established based on the literature [35,36]. The initial printing parameters were the same as for the basic composition and resin with ascorbic acid, and equaled 100 µm of layer height and 200 s of exposure time. During the printing process, under illumination by visible light, riboflavin is excited to a singlet state with a short life (10^−6^ s lifetime) followed by generation of a triplet excited state characterized by a long life (10^−2^ s lifetime) [37]. The triplet-excited riboflavin molecule accepts a proton from the triethanolamine, which forms α-amino radicals that attach to the monomers, increasing the rate of the free radical polymerization reaction [38]. As an effect, the addition of triethanolamine increased the surface of cured resin, which affected the diameter of the printed tablets; it was about 10% greater compared to the tablets printed at the same settings, without a co-initiator (Figure 6a,c and Table 6). Unlike the TP_AscA series, the TP_TRI_100/200 tablets had sharp and distinct boundaries despite the increased curing surface.

**Figure 7:**
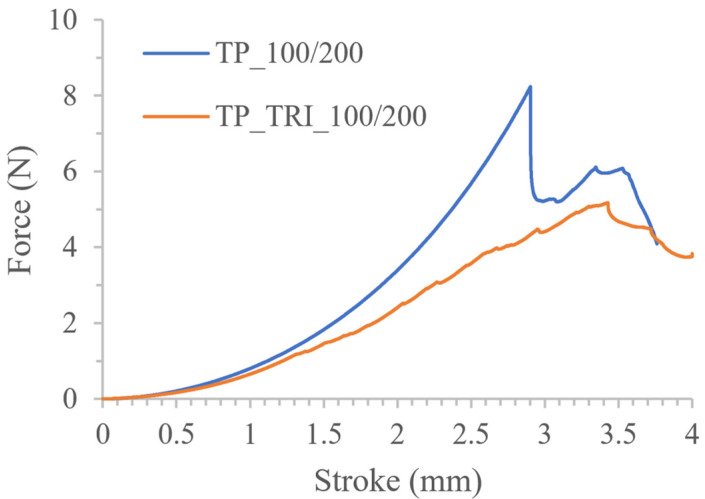
Comparison of the mechanical properties of tablets made of resin without co-initiator and with triethanolamine.

Differences in the properties of tablets made of resin with and without triethanolamine were also visible in the representative force-stroke curves, shown in Figure 7, determined in the texture analysis. Tablets printed with a resin without a co-initiator (TP_100/200) had a clear cut-off moment of penetration of the upper surface of the tablet by the testing probe at a force of approx. 8.40 N. A subsequent decrease in force indicated the tablet’s breakage. In the case of tablets containing triethanolamine (Figure 7, TP_TRI_100/200), the plot was irregularly shaped with no clear breakthrough moments in the tablet’s structure. This curve pattern may be a result of inhomogeneous resin curing, caused by insufficient light penetration; the resin cured during the printing of a single layer had a larger surface area than assumed in the design, but the curing process was not uniform throughout its height. Therefore, in the following stages of the experiment, we reduced the layer height to 50 µm to increase the homogeneity of the tablets’ structure. Moreover, we gradually reduced the time of exposure of the layers to increase the dimensional accuracy of the prints, i.e., to print tablets with a diameter of 10 mm.

The modifications of printing settings led to reductions in the mass and diameter of the TP_TRI_50/50 and TP_TRI_50/25 series of tablets (Table 6). Although the accuracy of the shape of the tablets from the project increased, and the tablets’ mass variability decreased, it still exceeded the pharmacopeial mass uniformity requirements. In the case of the subsequent series of tablets, there was a clear tendency to increase the mass of four tablets located in the central part of the printer’s platform, which may be directly related to the uneven exposure of the printer’s working area. Therefore, in the following tests, four tablets located in the middle part of the platform were not included. The externally located tablets were characterized by small deviations in mass and dimensions compared to the average calculated for the entire series of tablets.

Optimal printing parameters for placebo resin were determined at 50 µm layer height and 25 s exposure; the tablets had a diameter close to the theoretical one and repeatable hardness. Further reduction in the exposure time was unfavorable; in the case of TP_TRI_50/12.5 tablets, the exposure time was too short, as an effect, only eight tablets were printed. The total printing time for the TP_TRI_50/25 tablets was 80 min and was almost 60% shorter compared to the printing time for the TP_100/200 tablets (186 min).

**Figure 8:**
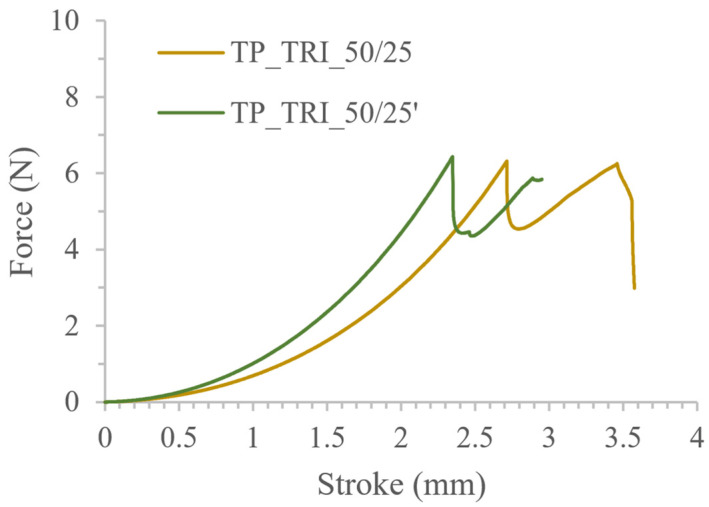
Comparison of the mechanical properties of tablets made of fresh and reused resin containing triethanolamine.

The influence of printing parameters on the tablets’ properties was also visible in the results of the mechanical tests. The course of the force-stroke curve of TP_TRI_50/25 tablets, presented in Figure 8, was similar to the TP_100/200 profile. The curve was initially smooth, with no roughness observed in the TP_TRI_100/200 tablets. With a force of approx. 6.5 N, the surfaces of the tablets were punctured and then broken. Furthermore, by adding triethanolamine to the resin, it was possible to reuse it for printing. The tablets from the second trial had almost the same mass, diameter, and similar mechanical properties; the maximum force value was comparable, but also, the shape of the force vs. stroke plot was almost the same (Table 6, Figure 8). A slightly steeper curve was obtained for tablets made of reused resin (TP_TRI_50/25′), which proved that those tablets were a bit more rigid. However, those small differences were found to be insignificant for the final quality of the printed tablets; thus, we confirmed the suitability of the reused resins.

### 3.4. Drug-Loaded Tablets

The first series of MEB-loaded tablets was printed with the optimal printing parameters for the placebo formulation, i.e., the layer height of 50 µm and 25 s of exposure time. The average mass of the TMEB_50/25 tablets, presented in Table 7, was approx. 20% lower compared to that of the TP_TRI_50/25 placebo tablets printed with the same printing parameters. The difference in mass between the placebo and drug-loaded tablets may be connected with the change in the resin composition; the addition of API caused a reduction in PEGDA concentration, thus reducing the resin’s ability to polymerize. Moreover, the diameter of the tablets was smaller compared to that of the placebo tablets and the project.

An attempt to reuse the resin remaining from printing the TMEB_50/25 tablets was considered unsuccessful; only nine tablets from the TMEB_50/25′ series were printed correctly. The remaining three broke off the printer’s platform during printing, another two had deformed edges. Therefore, we prepared a new drug-loaded resin with the same composition as that for printing the TMEB_50/25 tablets to repeat the tests with modified printing settings. The fresh resin was used to print tablets with increased exposure time equal to 50 s per one layer, marked as TMEB_50/50 tablets. The resin remaining after printing TMEB_50/50 tablets was mixed and used to prepare the TMEB_50/50′ tablets.

Extending the exposure time improved the quality of the tablets. Both series of tablets, made of fresh as well as reused resin, were printed completely. Moreover, there were no statistically significant differences in mass between the tablets from the TMEB_50/50 and TMEB_50/50′ series (*p* > 0.05). For both series of tablets printed at 50 s of exposure time, the mass deviation did not exceed ±5% from the mean value, i.e., the tablets met the requirements of the uniformity of mass test (2.9.5 *Uniformity of mass of single-dose preparations*) in accordance with Ph. Eur. [29]. According to the test, for tablets with an average mass equal to or greater than 250 mg, the mass deviation of each tablet should not exceed ±5%. Moreover, the content of mebeverine hydrochloride, presented in Table 7, in both series was almost the same. The measured drug loading of all tested series of tablets was slightly higher than the theoretical content of MEB in resin equal to 10%. This increase was probably caused by the water evaporation during the removal of the residual part of the resin and drying.

The extension of the printing time for MEB-loaded tablets from 25 to 50 s of exposure increased the force needed to break the tablets from approx. 6.4 N to over 7.7 N (Table 7, tablets TMEB_50/25 and TMEB_50/50, respectively). Moreover, MEB-loaded tablets printed at 50 s of exposure time had a 30% higher value for the force needed to break the tablets compared to the placebo tablets printed with the same parameters (series TP_TRI_50/50 and TMEB_50/50, presented in Table 6 and Table 7, respectively).

**Figure 9:**
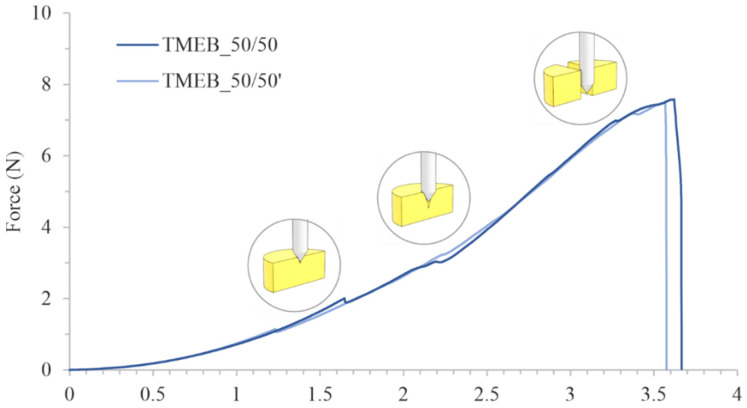
Comparison of the mechanical properties of drug-loaded tablets.

Both series of MEB-loaded tablets printed at 50 s of exposure time had an almost identical force-stroke curve and value for the force needed to break the tablets, as determined during the penetration test (Figure 9, Table 7 series TMEB_50/50 and TMEB_50/50′, respectively). The test started when the probe touched a tablet’s surface; the subsequent rise in the curve corresponded to the tablet’s elastic deformation. The courses of the curves were characterized by two small bends: one at a force of approx. 1.5 N, which was related to the puncture of the tablet’s surface by the needle probe, and another at approx. 2.0 N. The second bend was related to the needle probe cone base immersion into the tablet. Next, the tablets broke at a force of about 7.6 N. The differences between the values of the forces needed to break the TMEB_50/50 and TMEB_50/50′ tablets were not statistically significant (*p* > 0.05).

**Figure 10:**
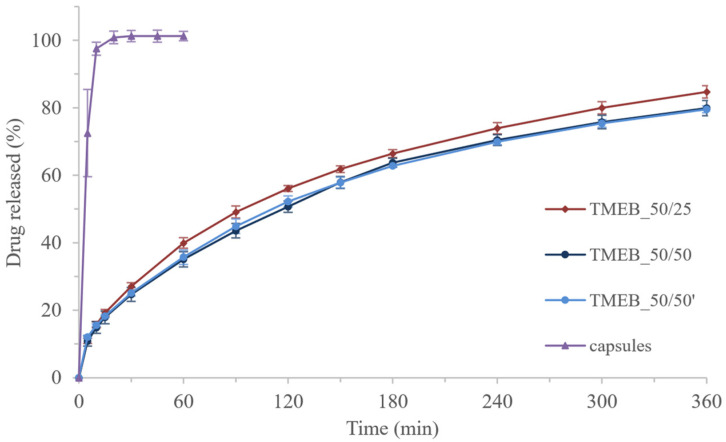
Dissolution profiles of MEB-loaded tablets.

Compounded drugs, such as compounded powders, are prepared in pharmacies when it is necessary to prepare a solid dosage form containing API doses adjusted to the patient’s needs. The preparation method includes filling the capsules with a mixture of powder made of raw substances or pulverized marketed solid dosage forms. Therefore, the drug release rate from such capsules depends mainly on the properties of the API, and the preparation of a sustained-release formulation is not possible. In contrast, the use of the photopolymerization process results in the formation of a polymeric matrix affecting the API release. While almost all of the mebeverine hydrochloride was released from the gelatin capsules within 10 min of the test, sustained release of API, reaching 80% after 6 h, was identified from 3DP tablets (Figure 10).

Mebeverine hydrochloride release profiles from 3DP tablets were almost the same during the first 30 min of the test. In the following hours, the amounts of API released from the TMEB_50/25 tablets were slightly higher compared to those printed with longer exposure times. To compare the dissolution profiles of MEB-loaded tablets, we calculated the similarity factor *f*_2_ (Equation (3)), which was used to measure the proximity of the two dissolution profiles. For two identical profiles, *f*_2_ = 100, whereas for profiles differing on average by 10% at all measured time points, *f*_2_ = 50.

(3) $$f_{2}=50\log\left\{{\left[1+\frac{1}{n}\sum_{n=1}^{n}{\left(R_{t}-T_{t}\right)}^{2}\right]}^{-0.5}\cdot 100\right\}$$

where *f*_2_ is the similarity factor, *n* is the number of observations, *R_t_* is the average percentage of drug dissolved from the reference formulation (TMEB_50/50), and *T_t_* is the average percentage of drug dissolved from the test formulation (TMEB_50/25 or TMEB_50/50′).

The difference between the series of tablets printed with different exposure times was not significant; the similarity factor calculated for the TMEB_50/25 and TMEB_50/50 series of tablets was equal to 70.63. In the case of tablets printed with an exposure time of 50 s, regardless of the resin used (new or reused), the release profiles were almost identical (*f*_2_ calculated for TMEB_50/50 and TMEB_50/50′ was equal to 94.31).

Extending the exposure time did not affect the mechanical properties or the API release profile from the printed tablets. However, it significantly improved the uniformity of the mass and the accuracy of the dimensions and also influenced the efficiency of the printing process, making it possible to reuse the resin.

By applying KinetDS software, available models were fitted to the dissolution curve of tablets printed with 50 s of exposure time, and the best results were obtained for the Korsmeyer–Peppas model (Equation (4)), for which R^2^ = 0.998 and RMSE = 1.86.

(4) $$Q=K\cdot t^{n}$$

where *Q* is the amount of drug released, *K* is constant, *t* is time, and *n* is release exponent.

The power law model might be applied to elucidate the drug release mechanisms from polymeric systems. It can be treated as a generalization of two apparently independent drug transport, diffusion, and relaxation mechanisms. Depending on the value of the release exponent (*n*) that better adjusts to the release profile of an active compound in a matrix system, it is possible to establish a drug-release mechanism. Regarding the shape of the dosage form (cylindrical tablet) and *n* value, we may distinguish four mechanisms of drug dissolution. The first one represents the Fickian diffusion model, in which the solvent transport rate or diffusion is much greater than the process of polymeric chain relaxation (*n* = 0.45). The next one, described by *n* = 0.89, represents the non-Fickian model, in which the drug release rate corresponds to zero-order release kinetics, whereas the mechanism driving the drug release is the swelling or relaxation of polymeric chains. In the case of 0.45 < *n* < 0.89, the model represents non-Fickian or anomalous transport, and the mechanism of drug release is governed by diffusion and swelling. The rearrangement of polymeric chains occurs slowly, and the diffusion process simultaneously causes the time-dependent anomalous effects. The last type of release mechanism, the Super Case II model, is highlighted for *n* exceeding 0.89. The process is characterized by tension and breaking of the polymer during the sorption of dissolution media [39].

**Figure 11:**
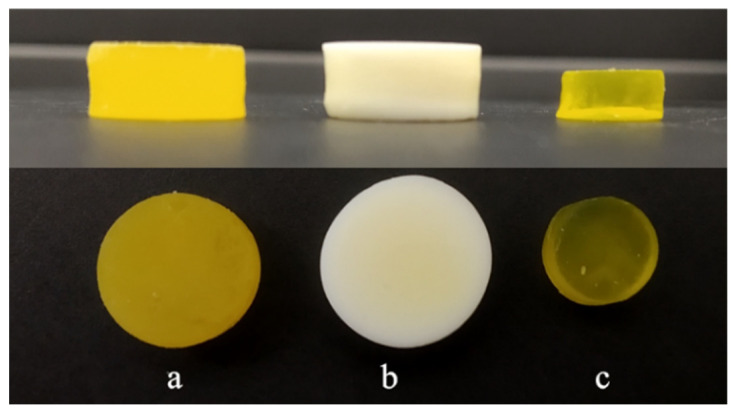
Side and top views of MEB-loaded tablets: after printing (**a**), directly after dissolution test (**b**), and after dissolution test and drying at room temperature (**c**).

According to the adopted procedure to properly estimate the release exponent value, the part of the dissolution curve with *Q* < 60% was applied. The obtained *n* value, equal to 0.48, implied that the drug release was represented by anomalous transport and both diffusion and swelling phenomena were involved [39]. An additional point supporting the participation of the swelling mechanism in the drug release was the fact that the dimensions of the tablets increased during the dissolution study. The matrix of tablets did not dissolve during the test but swelled: the tablet diameter measured directly after dissolution was approx. 10% greater than its original diameter (Figure 11). Moreover, after drying, the dimensions of the tablet were significantly reduced, which indicated the elastic nature of the polymer matrix remaining after the dissolution test.

### 3.5. Project Volume–Dosage Correlation

**Figure 12:**
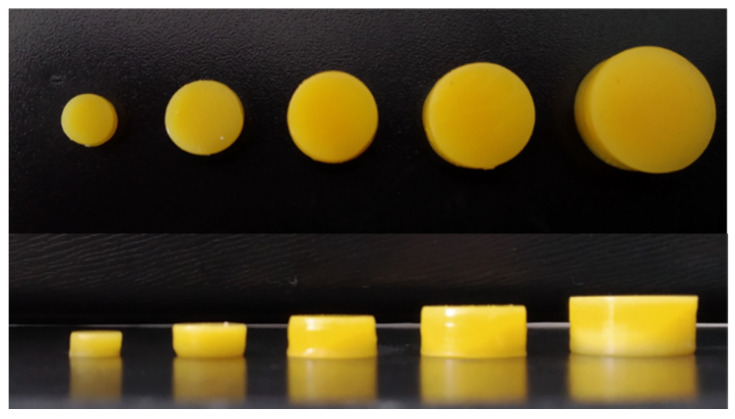
Top and side views of 3D printed MEB-loaded tablets with different volumes.

To confirm the possibility of printing tablets containing the assumed doses of the APIs, we designed, prepared, and analyzed printlets characterized by various volumes (Figure 12, Table 8).

**Figure 13:**
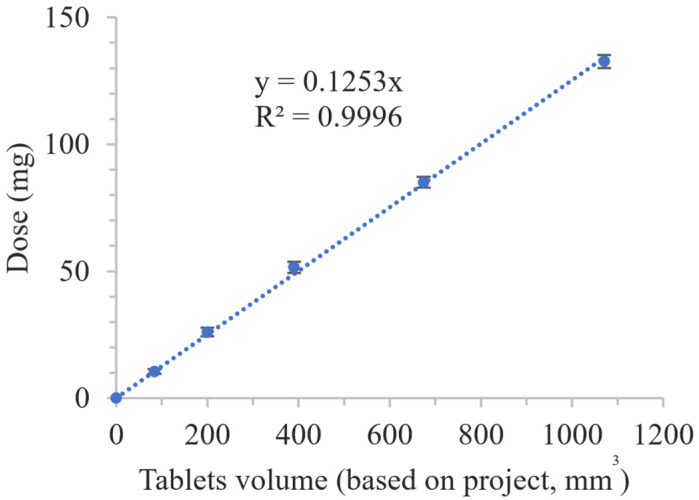
Volume–dose correlation.

All tablets were completely printed and were characterized by almost the same diameter as the project. Regardless of the dimensions of the tablets, the mean height for each size was, on average, 0.15 mm lower than assumed in the design. Despite the slight differences between the assumed and actual dimensions of the tablets, we determined a linear correlation between the volume of the printed tablets set at the stage of tablet design and the API dosages in the entire range of analyzed volumes (R^2^ = 0.9996, Figure 13). This dependence confirmed the possibility of quick adjustment of the design to print tablets containing the doses adjusted to the patient’s needs.

## 4. Conclusions

As a result of multi-stage modifications of the composition, printer structure, and process parameters, we developed an optimized placebo resin composition based on PEGDA 575 and PEG 400, containing a two-component photoinitiation system composed of riboflavin, a non-toxic photoinitiator. The addition of triethanolamine as a co-initiator shortened the printing time and influenced the stability of the resin. Therefore, it was possible to reuse the remaining uncured resin after printing. The optimized formulation was used for printing MEB-loaded tablets with uniform dosages, mechanical properties, and dissolution profiles for tablets made of fresh, as well as reused, resin. According to our knowledge, this is the first publication confirming the possibility of reusing drug-loaded resins left over from the previous process.

The presented results confirm the potential of the application of Daylight Polymer Printing technology to prepare small batches of personalized tablets characterized by modified-release of an active pharmaceutical ingredient. Due to the printer’s construction, the entire screen was exposed during the printing process, and the printing time did not depend on the number of printed tablets but only on their height. The resin preparation method requires only weighing and mixing the ingredients, e.g., using a magnetic stirrer, so that resin can be easily prepared before printing. Moreover, we confirmed the possibility of quick adjustment of the design to print tablets with a dose tailored to the individual needs of the patient.

## Figures and Tables

**Table 1:** The composition (% *w*/*w*) of the placebo resins based on the full factorial design of the experiment. Encoded levels of factors are presented in brackets.

Name	Tested Formulation Composition (% *w*/*w*)			
	PEGDA 575	PEG 400	Water	Riboflavin
P1	15.00 (−1)	79.90	5.00 (−1)	0.10
P2	45.00 (0)	49.90	5.00 (−1)	
P3	75.00 (+1)	29.90	5.00 (−1)	
P4	15.00 (−1)	69.90	15.00 (0)	
P5	45.00 (0)	39.90	15.00 (0)	
P5′	45.00 (0)	39.90	15.00 (0)	
P5″	45.00 (0)	39.90	15.00 (0)	
P6	75.00 (+1)	9.90	15.00 (0)	
P7	15.00 (−1)	59.90	25.00 (+1)	
P8	45.00 (0)	29.90	25.00 (+1)	
P9	75.00 (+1)	-	24.90 (+1)	

P5′, P5″—repetitions for P5 resin composition.

**Table 2:** Placebo tablet composition and printing parameters.

Name	Resin Composition (% *w*/*w*)					Printing Parameters	
	PEGDA 575	PEG 400	Water	Riboflavin	Additional Compound	Layer Height (µm)	Exposure Time (s)
TP_100/200	45.00	39.90	15.00	0.10	-	100	200
TP_50/100						50	100
TP_50/100′						50	100
TP_AscA1_100/200	45.00	38.90	15.00	0.10	AscA 1.00	100	200
TP_AscA2_100/200	45.00	39.40	15.00	0.10	AscA 0.50	100	200
TP_AscA3_100/200	45.00	39.80	15.00	0.10	AscA 0.10	100	200
TP_TRI_100/200	45.00	39.75	15.00	0.10	TRI 0.15	100	200
TP_TRI_50/50						50	50
TP_TRI_50/25						50	25
TP_TRI_50/25′						50	25
TP_TRI_50/12.5						50	12.5

′—tablets made of reused resin.

**Table 3:** Printer parameters for drug-loaded tablets.

Name	Layer Height (µm)	Exposure Time (s)
TMEB_50/25	50	25
TMEB_50/25′		
TMEB_50/50	50	50
TMEB_50/50′		

′—tablets made of reused resin.

**Table 4:** Mechanical properties of cured placebo resins, *n* = 5.

Name	Force (N) ± SD
P1	0.11 ± 0.02
P2	1.66 ± 0.23
P3	resin uncured
P4	0.11 ± 0.01
P5	2.05 ± 0.19
P5′	2.07 ± 0.44
P5″	2.13 ± 0.19
P6	0.98 ± 0.13
P7	0.09 ± 0.01
P8	1.00 ± 0.11
P9	resin uncured

P5′, P5″—repetitions for P5 resin composition.

**Table 5:** Comparison of first-order and second-order models fitted to experimental data. X1—PEGDA; X2—Water, Y—Hardness.

	First-Order Model	Second-Order Model
**Formula**	Y = C1 × X1 + C2 × X2 + Intercept	Y = C1 × X1 + C2 × X2 + C3 × X1 × X2 + C4 × X1^2^ + C5 × X2^2^ + Intercept
***p*-value**	0.9277	0.0046
**Coefficients**	Intercept = 0.9276 (*p* = 0.0159) C1 = 0.112 (*p* = 0.792) C2 = −0.115 (*p* = 0.788)	Intercept = 2.0294 (*p* = 5.94 × 10^−5^) C1 = 0.112 (*p* = 0.427) C2 = −0.115 (*p* = 0.417) C3 = 0.0057 (*p* = 0.973) C4 = −1.402 (*p* = 0.00091) C5 = −0.618 (*p* = 0.027)
**Residual standard error**	1.01	0.3179
**Multiple R-squared**	0.0186	0.9393
**Adjusted R-squared**	−0.2268	0.8786
**Shapiro–Wilk normality test of residuals**	0.1229	0.4518

**Table 6:** Characteristic of placebo tablets.

Name	Mass ^1^ (mg) ± SD	Diameter ^1^ (mm) ± SD	Force ^2^ (N) ± SD
TP_100/200	442.61 ± 41.60	10.81 ± 5.47	8.40 ± 0.79
TP_50/100	369.77 ± 49.98	9.90 ± 0.70	9.29 ± 0.73
TP_50/100′	Printing failed		
TP_AscA1_100/200	Printing failed		
TP_AscA2_100/200			
TP_AscA3_100/200			
TP_TRI_100/200	760.06 ± 103.19	12.08 ± 0.51	4.93 ± 3.43
TP_TRI_50/50	563.53 ± 55.58 537.62 ± 19.30 *	11.55 ± 0.64 11.26 ± 0.37 *	5.93 ± 0.57 *
TP_TRI_50/25	490.49 ± 28.50 477.15 ± 18.78 *	10.45 ± 0.23 10.38 ± 0.13 *	6.52 ± 0.26 *
TP_TRI_50/25′	487.67 ± 27.56 476.09 ± 17.88 *	10.32 ± 0.20 10.23 ± 0.08 *	6.28 ± 0.75 *
TP_TRI_50/12.5	Printing failed		

^1^*n* = 18, ^2^ *n* = 6, * Evaluated only for external tablets (*n* = 14); ′—tablets made of reused resin.

**Table 7:** Characteristics of MEB-loaded tablets.

Name of Tablets	Mass ^1^ (mg) ± SD	Diameter ^1^ (mm) ± SD	Force ^2^ (N) ± SD	Drug-Loading ^3^ (%) ± SD	Dosage (mg)
TMEB_50/25	388.39 ± 13.71	9.78 ± 0.17	6.44 ± 0.66	10.83 ± 0.32	41.94
TMEB_50/25′	384.06 ± 17.41 *	9.41 ± 0.30 *	-	-	-
TMEB_50/50	423.66 ± 9.96	10.12 ± 0.19	7.73 ± 0.53	11.21 ± 0.02	47.73
TMEB_50/50′	426.66 ± 5.79	10.02 ± 0.11	7.60 ± 0.26	11.00 ± 0.17	47.23

^1^*n* = 14, ^2^ *n* = 6, ^3^ *n* = 3, * only 9 of 14 tablets printed correctly, ′—tablets made of reused resin.

**Table 8:** Parameters characterizing the designed and printed tablets (*n* = 3).

Project			3D Printed Tablets		
Diameter (mm)	Height (mm)	Volume (mm^3^)	Diameter (mm) ± RSD (%)	Height (mm) ± RSD (%)	Mass (mg) ± RSD (%)
14	7	1070.66	14.01 ± 0.51	6.72 ± 0.43	1194.70 ± 1.59
12	6	674.23	12.01 ± 0.34	5.72 ± 0.49	766.16 ± 2.00
10	5	390.18	10.13 ± 0.05	4.80 ± 0.77	464.35 ± 3.59
8	4	199.77	8.03 ± 0.20	3.77 ± 0.87	234.54 ± 5.31
6	3	84.28	5.94 ± 0.95	2.77 ± 1.68	94.66 ± 7.56

## Data Availability

Not applicable.
